# Evaluating the effectiveness of biotic indices for long-term ecological quality assessment in a heavily polluted estuary

**DOI:** 10.1007/s10661-025-14546-w

**Published:** 2025-09-10

**Authors:** J. Emilio Sánchez-Moyano, Mario López-Cepeda, Isabel García-Asencio

**Affiliations:** Department of Zoology, Faculty of Biology, University of Sevilla, Av. Reina Mercedes 6, 41012 Seville, Spain

**Keywords:** Heavy metal, Ecological monitoring, Subtidal communities, Odiel Estuary, South-West Spain

## Abstract

Marine ecosystems, particularly estuaries, are increasingly threatened by anthropogenic pressures. The Odiel Estuary has suffered severe contamination from acid mine drainage and industrial activities. Since 1986, mitigation efforts have been implemented, yet their long-term ecological effectiveness remains under-evaluated. This study assesses environmental recovery by analyzing six biotic indices over 18 years (1998–2016). Our objectives were to determine spatial gradients and temporal improvements in ecological status within the estuary using these indices, and to identify the most reliable monitoring tools for transitional ecosystems. Results revealed a clear spatial gradient, with inner estuary sites in poor condition and outer marine zones showing better status. A detectable improvement in benthic community structure and water quality was observed over time, particularly in 2016. M-AMBI and BENFES correlated strongly with species diversity and effectively captured environmental gradients, whereas AMBI, BENTIX, and BOPA/BO2A showed lower sensitivity. Despite observed improvements, persistent heavy metal contamination requires continued monitoring and management. The study underscores the challenge of distinguishing natural estuarine variability from anthropogenic impacts, advocating for multi-index approaches to enhance ecological assessments. These findings provide insights for future monitoring and standardized protocols in transitional waters globally.

**Supplementary Information:**

The online version contains supplementary material available at 10.1007/s10661-025-14546-w.

## Introduction

Marine ecosystems are sustained by the energy flow among various organisms, from primary producers to top predators, forming complex biological networks (Doney et al., 2012). These ecosystems play a crucial role in providing essential services to human society, such as nutrient recycling, carbon capture and storage, pollutant detoxification, coastal erosion mitigation, food production, and cultural activities (Lu et al., 2018; Parsons et al., 2014). For these reasons, coastal areas have undergone significant development and exploitation over recent decades, accompanied by a substantial increase in human population density (Defeo et al., 2009; Neumann et al., 2015). Currently, more than 3000 million people live in coastal regions, posing a serious environmental threat (Martínez et al., 2007; Santibañez-Aguascalientes et al., 2023). This issue is particularly critical in estuarine environments, where human activities have already imposed severe pressures, including urbanization, agricultural runoff, overfishing, intensive aquaculture, and industrial pollution. These factors contribute to habitat degradation, biodiversity loss, and water quality deterioration (Donázar-Aramendía et al., 2019; Kennish, 2002; Kennish et al., 2014; Korpinen et al., 2012). Furthermore, these negative effects are expected to intensify in the future due to climate change-related factors (Cloern et al., 2016; Elliott et al., 2015; Newton et al., 2013).

Monitoring is essential for tracking changes in water quality and biodiversity, which serve as key indicators of ecosystem health (Elliott & Whitfield, 2011; Lipcius et al., 2019). Addressing this challenge requires a holistic approach that integrates scientific expertise, stakeholder engagement, and adaptive management strategies (Barbier, 2013; Birk et al., 2012; Borja et al., 2008; Goulding et al., 2021; Spalding et al., 2007).

In this context, the European Union developed the Water Framework Directive (WFD 2000/60/EC) and the Marine Strategy Framework Directive (MSFD 2008/56/EC) to establish a unified framework for water policy, aiming to maintain and improve aquatic ecosystem health. These directives require that, to ensure comparability in water body assessments, indicators must be expressed as quality indices based on biological parameters. The WFD sets emission limits through specific mass or concentration values for particular parameters, which are then used to determine the ecological quality status (EQS) of a given area. A key component of this assessment is the concept of reference conditions, which represent the expected biological and physicochemical state of a water body under minimal or no human impact. These conditions provide a baseline for evaluating ecological degradation and recovery. Both directives mandate that member states assess the ecological quality status of coastal and transitional waters, aiming to achieve a “good status” for European water bodies. If this status is not met, appropriate measures must be implemented to restore conditions similar to ecological reference states (Feebarani et al., 2016). In recent years, these directives have been complemented by broader EU policy instruments, such as the EU biodiversity strategy for 2030 and the Nature Restoration Law. While the biodiversity strategy outlines non-binding targets to reverse biodiversity loss and expand protected areas, the Nature Restoration Law, adopted in 2024, introduces legally binding obligations to restore at least 20% of degraded terrestrial and marine ecosystems by 2030. Both initiatives reinforce the implementation of the WFD and MSFD by promoting ecological restoration, improving habitat connectivity, and enhancing the resilience of aquatic ecosystems. Collectively, these instruments reflect a policy shift from monitoring and assessment toward active, ecosystem-based management and large-scale ecological restoration.

These environmental policies, along with growing public awareness, have driven the development of various methods for assessing environmental quality, including biotic indices (Borja et al., 2015; Rosado et al., 2015). Most of these indices are based on the composition of benthic communities due to their advantages, such as ease and low cost of sampling, high species diversity with various biological traits, low mobility and sufficient longevity, their key role in food web productivity and nutrient cycling, and their sensitivity to both anthropogenic and natural stressors (Dash et al., 2021; Goulding et al., 2021; Kaboré et al., 2016). These characteristics allow benthic communities to provide functional responses to different environmental disturbances, making them useful for ecological status assessment (Neves et al., 2025; Paul et al., 2023).

The use of biotic indices has been considered an effective tool for evaluating marine ecosystem health and has been tested across various habitats and different levels of human disturbance in different geographic regions (Dauvin et al., 2012; Pinto et al., 2009). The reliability and usefulness of the biotic indices depend on their accuracy, precision, generality, and robustness (Basset et al., 2012). However, numerous criticisms have emerged regarding their applicability (Karydis, 2022), arguing that they may oversimplify ecological systems (Aguado-Giménez et al., 2015; Oprandi et al., 2023) or that no single index can be considered universally reliable, as each has been developed for specific stressors, communities, or sites (Afli et al., 2008; Mulik et al., 2020; Muniz et al., 2012; Pinto et al., 2009). Another major challenge is distinguishing between natural and anthropogenic disturbances (Elliott & Quintino, 2007), particularly in estuaries (Dauvin & Ruellet, 2009). These naturally stressed systems, characterized by frequent environmental fluctuations (Chilton et al., 2021; Mitchell et al., 2015), often host communities with high resistance to pollution, making the interpretation of disturbance effects particularly challenging (Dauvin, 2008; Tweedley et al., 2015). This phenomenon, commonly referred to as the *Estuarine Quality Paradox*, highlights the difficulty of discerning whether observed ecological conditions result from natural variability or human-induced impacts—an issue that complicates the application and interpretation of biotic indices in transitional waters. As a potential solution, many authors recommend the combined use of multiple indices that provide complementary information for assessing environmental quality (Dauvin et al., 2012; Partha et al., 2024; Salas et al., 2006; Teixeira et al., 2010). Furthermore, indices should be validated across various systems to determine which respond most effectively to different stressors (Mulik et al., 2020).

The Odiel Estuary, located in southwestern Spain, is formed by the confluence of the Tinto and Odiel rivers. The watersheds of both rivers are situated in the Iberian Pyrite Belt (IPB), one of the world’s largest polymetallic sulfide deposits (Barba-Lobo et al., 2024), which has been exploited since Phoenician and Roman times (Ortiz-Mateo, 2004). Consequently, these watersheds are affected by acid mine drainage, resulting in high metal concentrations (Nieto et al., 2013; Sarmiento et al., 2009). Additionally, the lower estuary is home to significant population centers, particularly the city of Huelva (~ 150,000 inhabitants), which has experienced intensive industrial and port activity since the 1960 s (Millán-Martínez et al., 2021). Major industries include phosphate fertilizer plants, an oil refinery, power plants, and other chemical industries (Barba-Lobo et al., 2024; Pérez-López et al., 2011, 2016; Ruiz, 2001).

Since 1986, the regional government has implemented corrective measures, including mine closure management, reduction of industrial and urban waste, and enhanced water pollution control (Ramos-Martín, 2005; Usero et al., 2000). Although local improvements in water quality and contamination levels have been reported from a chemical and geological perspective (Bonnail et al., 2019; Pérez-López et al., 2011; Sáinz et al., 2003), the effectiveness of these measures from a biological and ecological standpoint remains poorly studied, except through traditional macrobenthic community analyses (Sánchez-Moyano et al. 2010; Sánchez-Moyano & García-Asencio, 2010; Sánchez-Moyano et al., 2025).

In this context, the main objective of this study was to evaluate the long-term effectiveness of corrective measures on the environmental quality of the Odiel Estuary using a comparative analysis approach of six biotic indices. Our hypotheses were: 1) the spatial natural physicochemical gradient along the estuary should be reflected in the ecological status established by the different indices; 2) any changes in environmental conditions following the implementation of corrective measures should also be reflected in the ecological status according to the sensitivity of each index. Furthermore, the ultimate goal of this study was to identify which among the six benthic indices responded most effectively to these questions in order to develop a simplified monitoring routine for assessing the ecological quality of this or similar transitional systems worldwide.

## Material and methods

### Study area

This well-mixed mesotidal estuary is formed by the confluence of the Tinto and Odiel rivers over approximately 25 km, encompassing around 15,000 ha of salt marshes (Ruiz et al., 1998), which are recognized as an important naturally protected area, particularly for bird populations (Sánchez-Moyano et al., 2025). The estuary opens into a navigable channel (Padre Santo Channel) that extends southeast for 13 km to the Atlantic Ocean. During summer, this estuary experiences strong evaporation and limited freshwater input, behaving like an inverse estuary (Amaral et al., 2020), although this pattern is more evident upstream, outside the study area.

### Sampling and laboratory analysis

**Fig. 1:**
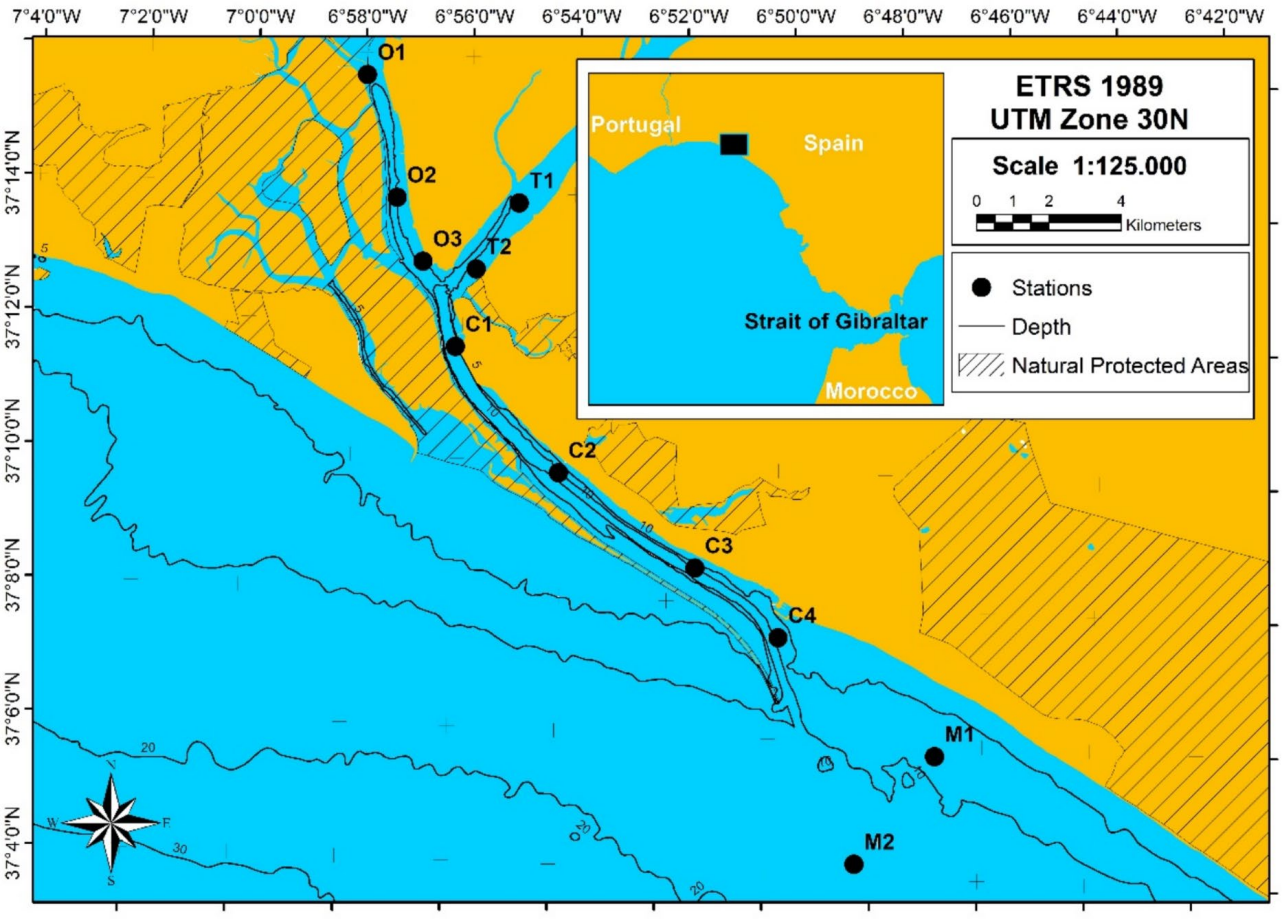
Location of the sampling stations in the Odiel–Tinto estuary. Stations were grouped into four zones: Inner estuary (stations O1 to O3 and T1 to T2), Inner Channel (stations C1 and C2), Outer Channel (stations C3 and C4), and Marine Area (stations M1 and M2)

Sampling was carried out during the ebb tide in the summer of 1998, 2000, 2002, and 2016 at 11 subtidal stations: 3 in the Odiel River, 2 in the Tinto River, 4 in the Padre Santo Channel, and 2 in the near coastal area (Fig. 1). Both marine stations were missing in the 1998 sampling event, and station T1 (upstream in the Tinto River) was added in 2016. The stations were grouped into four zones: Odiel or inner estuary (points from Odiel –O1 to O3– and Tinto –T1 and T2–), Inner Channel (stations C1 and C2), Outer Channel (stations C3 and C4), and Marine Area (stations M1 and M2). These areas, although initially defined based on their environmental characteristics, were also established by previous studies of benthic communities in the zone (see, for example, Sánchez-Moyano et al. 2010; Sánchez-Moyano & García-Asencio, 2010; Sánchez-Moyano et al., 2025). All sampling campaigns were conducted during the dry season, which corresponds to the period of greatest benthic community development in temperate estuarine systems, due to higher biological productivity and more stable environmental conditions (Chainho et al., 2007). This temporal consistency was selected to ensure comparability across years and to capture the communities at their peak structural maturity. However, this approach does not account for potential seasonal variations, which are known to influence biological dynamics in estuarine systems. Therefore, the absence of intra-annual sampling may limit the interpretation of short-term temporal variability.

At each station, six replicate samples (five for biological analysis and one for sediment analysis) were taken using a 0.05 m^2^ van Veen grab. Each macrofauna sample was sieved in seawater through a 0.5 mm mesh and fixed in ethanol (70%), stained with Rose Bengal for subsequent identification and quantification to the species level whenever possible. Scientific names were verified with the World Register of Marine Species (WoRMS http://www.marinespecies.org).

For sediment analysis, organic matter was obtained as weight loss by ignition at 500 °C for 4 h (mean value of 3 replicates per station), and granulometry was evaluated following Buchanan and Kein (1984). Total organic carbon (TOC) was determined by EPA 415.1. For heavy metal concentrations (Cd, Zn, Cu, Cr, and Hg), sediments were taken from the uppermost 2 cm. In the laboratory, sediment samples were air dried, crushed, and sieved through a 2 mm sieve, and then ground to < 60 µm. These samples were digested with aqua regia (1:3 conc. HNO3: HCl) in a microwave digester. Quantification of elements in the extracts was achieved using a VARIAN ICP 720-ES (simultaneous ICP-OES with axially viewed plasma). The accuracy of the analytical methods was assessed using a reference soil sample from Wageningen Evaluating Programmes for Analytical Laboratories for soils, International Soil-analytical Exchange (WEPAL; ISE). The index of geoaccumulation (*I*_geo_) was used as a relative measure of metal pollution in the sediments for Cr, Cu, and Zn according to the regional background established by Ruiz (2001) for unpolluted sandy and silty–clayey sediments. It is given by *I*_geo_ = log_2_ (Cn/1.5 9 Bn), where Cn is the value of the element n, and Bn is the background data of that element. The index values were divided into five groups: unpolluted (*I*_geo_ < 1); very low polluted (1 < *I*_geo_ < 2); low polluted (2 < *I*_geo_ < 3); moderate polluted (3 < *I*_geo_ < 4); highly polluted (4 < *I*_geo_ < 5); very highly polluted (*I*_geo_ > 5). Unfortunately, nutrient concentration data were not available for the study period, which limits the ability to fully assess the relative contribution of eutrophication to the benthic community patterns. Given that nutrient enrichment is a key stressor in estuarine environments, future monitoring programs should prioritize its inclusion to improve the interpretation of ecological responses alongside other stressors such as metal contamination.

The water parameters were analyzed from a water sample of the bottom layer using a vertical Alpha Van Dorn-style bottle. All parameters were measured in situ: temperature and salinity by conductivity meter WTW LF-323; pH by pH meter WTW 330i; and dissolved oxygen by oximeter WTW OXI-196. In the 2016 sampling event, a Eureka Manta 2 multiparametric probe equipped with sensors for depth, pH, dissolved oxygen, and salinity was used.

### Biotic indices

Firstly, macrofauna data were analyzed to obtain the total number of taxa, abundance (as ind/m^2^), and Shannon diversity index (*H*′ based on log *e*). Macrofauna abundance data were used to determine the ecological status according to the intervals of six biotic indices shown in Table 1.

**Table 1:**
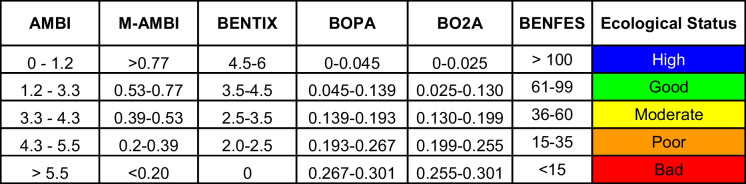
Intervals for establishing the ecological quality status according to the six biotic indices used

AMBI (AZTI marine biotic index) (Borja et al., 2000) is widely used in the WFD to determine the ecological status and is based on the assignment of species to five ecological groups (EGs) related to their degree of sensitivity and/or tolerance to environmental stress: most sensitive species (EG I), species indifferent to disturbance (EG II), species tolerant to disturbance (EGIII), second order-opportunistic species (EG IV), and first-order opportunistic species (EG V) (Borja et al., 2015).

M-AMBI is based on a factorial analysis which includes AMBI, Shannon diversity index (H′ based on log_2_) and richness (Muxika et al., 2007). The establishment of reference conditions in each environmental area is necessary to calculate M-AMBI. Due to the absence of suitable pristine areas within the Odiel-Tinto estuarine system, reference conditions were established based on several studies of soft-bottom communities in the lower estuary and in nearby coastal areas of the Guadiana River (Donázar-Aramendía et al., 2019; Sánchez-Moyano & García-Asencio, 2011; Sánchez-Moyano et al., 2017). The Guadiana and Odiel-Tinto estuaries are located in a Mediterranean climate region and both discharge into the Gulf of Cádiz along the Atlantic coast, less than 50 km apart, within the Iberian Pyrite Belt. These estuaries are mesotidal and vertically well-mixed, with low summer flow and episodic freshwater runoff during winter (González-Ortegón et al., 2015; Wolanski et al., 2006). Unlike the Odiel-Tinto, the Guadiana estuary is subject to relatively lower anthropogenic pressure (Donázar-Aramendía et al., 2019; Sánchez-Moyano et al., 2017) and has been described as one of the least polluted estuaries in Europe (Vasconcelos et al., 2007), although it flows through a predominantly rural area influenced by agriculture, agro-industrial activities, and dam regulation (Sánchez-Moyano & García-Asencio, 2011). Moreover, it is considered one of the best-preserved and most ecologically vulnerable estuaries on the Iberian Peninsula (Barbosa et al., 2010). AMBI for high status was calculated by removing the species of ecological groups IV and V from the dataset, according to Muxika et al. (2007). Due to estuaries showing a clear environmental gradient that can determine the composition of benthic communities, two reference conditions were established for high ecological status. For internal areas (Odiel and Inner Channel), AMBI 1.3, diversity 3.8, and richness 35. For external areas (Outer Channel and Marine Area), AMBI 1.3, diversity 4, and richness 50. Reference conditions for bad status were considered upon an azoic environment (AMBI = 6; richness and diversity = 0). Both indices were calculated using AMBI 6.0 software and the AMBI species List available in May 2022 (in cases of non-assigned species or discrepancies with AMBI categories, species were included in a level according to our experience or prior literature).

BENTIX uses ecological groups similar to the AMBI index, with the exception that, instead of using five groups, it is based on three broader ecological groups, thus avoiding uncertainty in the ecological group assignment procedure (Simboura & Zenetos, 2002). According to Simboura and Argyrou (2010), group I includes species sensitive to disturbances (though, it also includes species indifferent); group II includes species tolerant to disturbances (may also include second-order opportunistic species); group III consists of first-order opportunistic species.

BOPA (Benthic Opportunistic Polychaetes Amphipods) (Dauvin & Ruellet, 2007) and BO2A (Benthic Opportunistic Annelids Amphipods) (Dauvin & Ruellet, 2009) are based on the relative frequencies of opportunistic polychaetes or opportunistic annelids, respectively, and amphipods (except for *Jassa* species).

BENFES (Benthic Families Ecological Status Index) is based on presence/absence and identification at the family level (Sánchez-Moyano et al., 2017). Each family’s value is determined based on the pollution tolerance levels of its constituent species, following an inverse scale from AMBI, where more sensitive families are assigned a value of 5 and more tolerant families a value of 1. For families with species spanning different tolerance categories, the assigned value was based on the most abundant species or, in cases with multiple co-dominant species, the average value was applied.

As summary, the main characteristics of the six biotic indices used are shown in Table 2.

**Table 2:** General characteristics of the six biotic indices used

Index name	Taxonomic level	Data type	Ecological groups	Target animal group	Reference conditions required
AMBI	Species	Abundance	5	Macrobenthos	No
M-AMBI	Species	Abundance	5	Macrobenthos	Yes
BENTIX	Species	Abundance	3	Macrobenthos	No
BOPA	Species	Abundance	Opportunistic polychaetes	Amphipods + polychaetes	No
BO2A	Species	Abundance	Opportunistic annelids	Amphipods + annelids	No
BENFES	Family	Presence/absence	5	Macrobenthos	No

### Data analysis

Sediment and water variables (previously normalized) were examined using principal component analysis (PCA). Affinities between areas and sampling periods were assessed using nMDS (non-metric multidimensional scaling) analysis based on square-root-transformed species abundance data.

Spatio-temporal differences in univariate and multivariate datasets were tested using permutational analysis of variance (PERMANOVA; Anderson, 2001) with 9999 permutations based on Euclidean distance similarity matrices (for univariate and environmental variables) or Bray–Curtis dissimilarity matrices (for species abundance data). Significant results were followed by post hoc pairwise comparisons to identify sources of differences. These univariate and multivariate analyses were performed with PRIMER-E v6.1 and PERMANOVA (PRIMER-E Ltd.) (Anderson et al., 2008; Clarke & Gorley, 2006).

The relationships between indices were determined by the Pearson correlation. Canonical correspondence analysis (CCA) (Ter Braak, 1986, 1990) was carried out to establish the relationships among the biotic indices, the environmental characteristics, and the composition of the communities over the zones and sampling events. The variation inflation factor (VIF) was used as a criterion to select environmental variables in the model and avoid collinearity (variables with VIF > 10 were excluded). The statistical significance of the analysis was evaluated using Monte Carlo permutation tests. A permutation test to evaluate the individual contribution of each selected variable and the biotic indices was also performed. Pearson correlation and CCA were carried out using the BiodiversityR v. 2.16–1 package in R (Kindt & Coe, 2005).

An inter-rater reliability analysis using the Kappa statistic was performed to determine consistency among EQS obtained by the different indices for each zone. Since data are ordinal and possible misclassification is more important between close categories than between distant categories, we carried out a weighted Kappa (Fleiss & Cohen, 1973) using the package “irr” v. 0.84 (Gamer et al., 2015) in R. The level of agreement between indices was established based on the equivalence table from Monserud and Leemans (1992).

## Results

### Environmental parameters

The means of the water and sediment parameters over the sampling periods and zones are shown in Table S1. The Odiel-Tinto system is characterized as a euhaline estuary, and the water parameters exhibited typical estuarine trends across all periods, such as increasing pH and dissolved oxygen. However, a slight decrease in salinity was observed from inner areas to the river mouth. Sediment granulometry showed a predominance of fine elements (silts and clays) in the inner area and sands toward the outer channel and the marine area regardless of sampling period. Similarly, the percentage of organic matter was higher in the inner areas (range 5.8–13.3) than in the outer ones (range 0.2–4.4). Heavy metal concentrations showed a decreasing trend toward the mouth, with very high values in the inner zones for metals such as Zn and Cu (maximum of 3836.5 and 1925.3 mg/kg, respectively). In this context, the geoaccumulation index (*I*_geo_) for Zn and Cu showed high levels of contamination in the sediment in all areas and years, with the exception of the marine area, which showed moderate or slightly contaminated conditions, reaching a minimum level of contamination with respect to Cu in 2016. The *I*_geo_ for Cr indicated unpolluted or very slightly polluted sediment in most areas and periods.

**Fig. 2:**
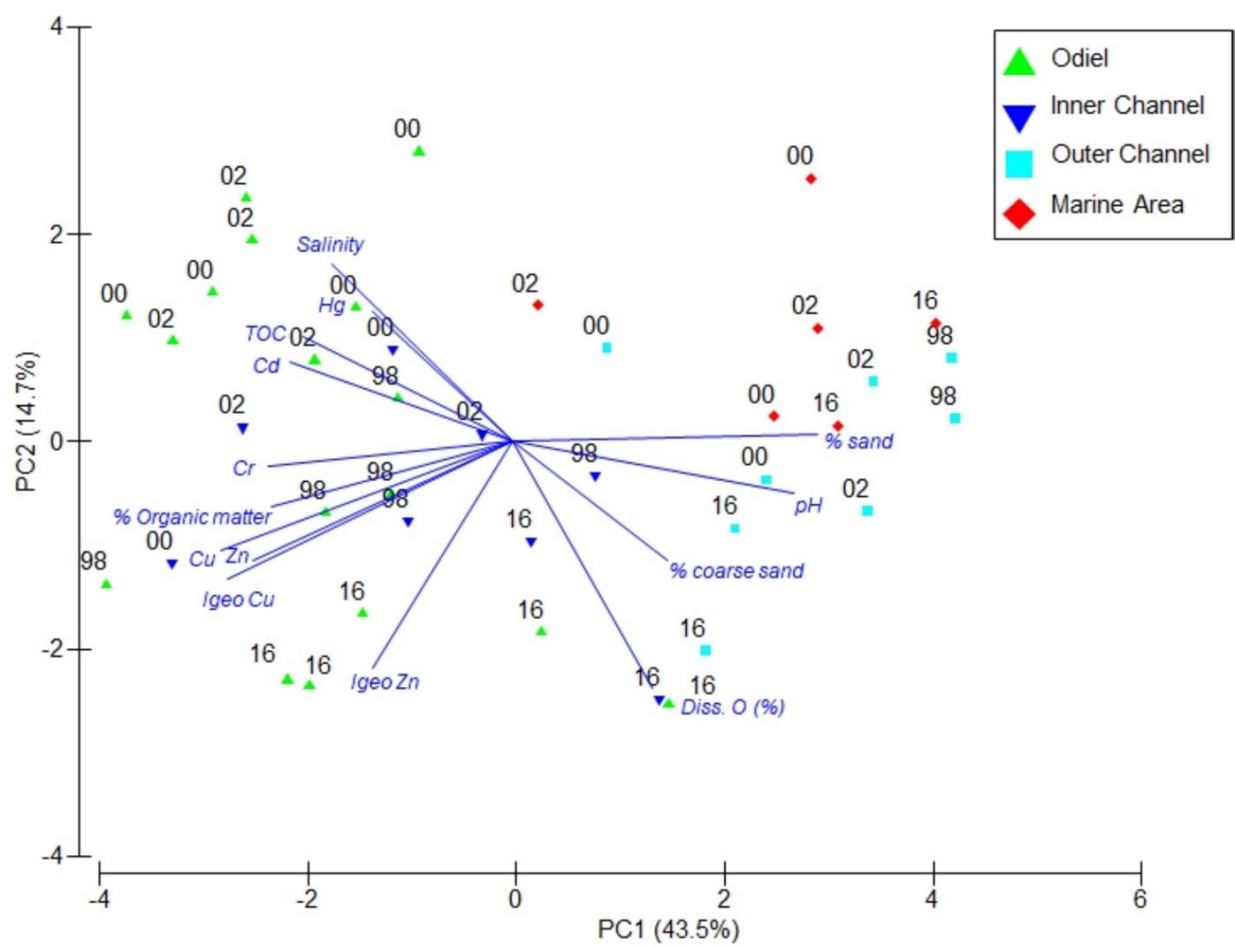
PCA plot for all stations and sampling periods based on water and sediment parameters. The percentage of variability explained by the two principal axes is given

PCA analysis of selected water and sediment parameters is shown in Fig. 2. The first two principal axes retained 58.2% of the variance. Axis one distinguished areas along the natural estuarine to marine gradient, as stated by the eigenvectors, % of sands (0.36), pH (0.33), Cu (− 0.34), and Zn (− 0.30). Axis two primarily distinguished between 2016 and the rest of the sampling events, especially in the inner areas, influenced by dissolved oxygen (0.50), salinity (− 0.36), and *I*_geo_ for Zn (− 0.46).

A multivariate PERMANOVA test based on Euclidean distances showed significant spatial and temporal differences, but not interaction (Table 3). Spatial pairwise comparisons indicated two homogeneous groups: Odiel and Inner Channel; and Outer Channel and Marine Area. From a temporal point of view, significant differences were detected in all years, except between 1998 and 2002 and between 2000 and 2002.

**Table 3:** PERMANOVA and pairwise test results based on the Euclidean distance matrix of the standardized environmental variables. In bold, significant results

Source	Df	SS	MS	Pseudo-*F*	*p*	Unique perms
Zone	3	198.53	66.18	9.11	**0.001**	9939
Year	3	68.06	22.69	3.12	**0.001**	9901
ZoxYe	8	77.12	9.64	1.33	0.07	9865
Residuals	24	174.31	7.26			
Total	38	532				
Pair-wise tests	*t*	*p*	Unique perms			
Zones						
Odiel, Inner Channel	1.43	0.059	9939			
Odiel, Outer Channel	4.28	**0.000**	9934			
Odiel, Marine	3.56	**0.000**	9936			
Inner and Outer Channel	2.76	**0.003**	9908			
Inner Channel, Marine	2.45	**0.006**	9943			
Outer Channel, Marine	1.40	0.124	9946			
YEARS						
1998, 2000	1.64	**0.016**	9948			
1998, 2002	1.55	**0.047**	9943			
1998, 2016	1.96	**0.003**	9961			
2000, 2002	1.08	0.309	9953			
2000, 2016	2.03	**0.001**	9930			
2002, 2016	2.20	**0.001**	9937			

### Macrofauna analysis

A total of 181 species were found throughout the study area during the four sampling events, belonging to 10 phyla: Arthropoda (81), Annelida (50), Mollusca (39), Echinodermata (3), Chordata (2), Cnidaria (2), Phoronida (1), Platyhelminthes (1), Chaetognatha (1), and Nemertea (1). The mean abundance for each zone and sampling is presented in Table S2. Dominant species in each area reflected the spatial gradient observed. In the Odiel area, the most dominant species throughout the study period was the polychaete *Streblospio shrubsolii*, along with other polychaetes such as *Polydora hoplura*, *Hediste diversicolor*, and *Nephtys hombergii*, as well as the crustaceans *Cyathura carinata*, *Monocorophium acherusicum*, and *Leptocheirus pectinatus*, and the bivalve *Cerastoderma edule*. The Inner Channel was also dominated by *S. shrubsolii*, although in lower abundance compared to the more internal zones. Other common annelids included *N. hombergii*, *Aonides oxycephala*, *Lagis koreni*, and oligochaetes, while crustaceans were less represented, with the exception of the isopod *C. carinata*. The Outer Channel was characterized by a higher number of species, with the bivalves *Spisula subtruncata* and *Chamelea gallina* being dominant, along with other mollusks such as *Varicorbula gibba* and *Donax trunculus*. Polychaetes were represented by species like *Scoloplos typicus*, *N. hombergii*, *Sigambra parva*, and *Glycera tridactyla*. Crustaceans were less abundant and included *M. acherusicum* and *Chondrochelia dubia*. Finally, the Marine area was also characterized by high species richness, with polychaetes such as *Mediomastus capensis*, *Prionospio steenstrupi*, and *Magelona papillicornis* being clearly dominant, along with mollusks like *Ouardia compressa*, *V. gibba*, *S. subtruncata*, and *C. gallina*, as well as crustaceans of the genera *Ampelisca* and *Iphinoe*, and the echinoderm *Echinocardium cordatum*.

**Fig. 3:**
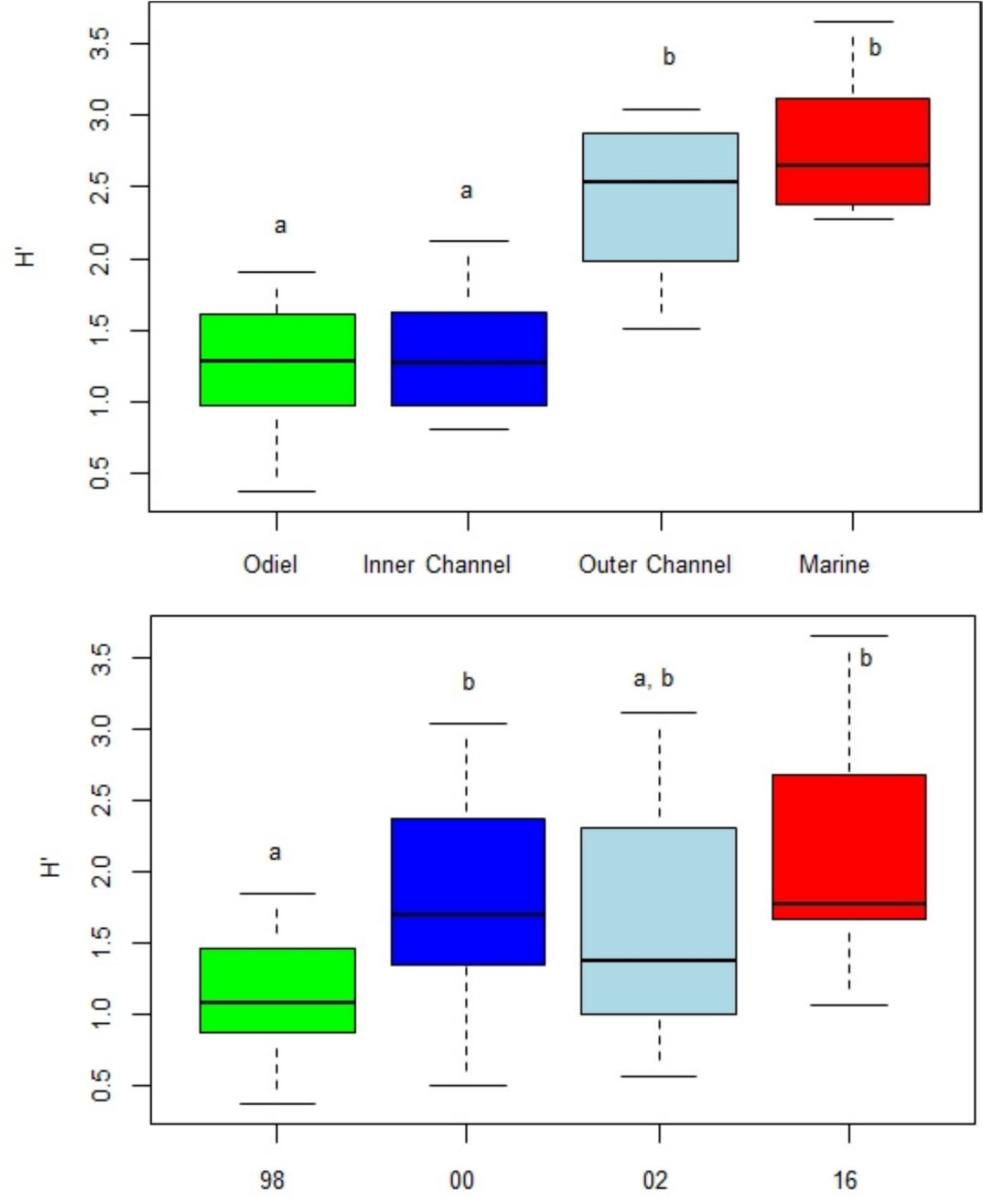
Box plots of the Shannon–Wiener diversity index (*H*′) along the study area and sampling events. The thick line corresponds to the median; the rectangles contain 50% of the values, between the 1 st and 3rd quartiles; the thin lines connect the extreme values, unless located at a distance greater than 3 times the height of the rectangle, in which case they are indicated by a circle. Homogeneous groups (*p* > 0.05) between zones and/or samplings are represented by different lowercase letters

Shannon diversity index exhibited a trend of increase toward the channel mouth and marine areas, with minimal temporal changes (Fig. 3). PERMANOVA analysis revealed significant spatial and temporal differences, but no interaction effects (Table 4). Pairwise comparisons identified two homogeneous groups: Odiel and Inner Channel; and Outer Channel and Marine. From a temporal perspective, 2016 showed higher values, though the only significant differences were found between 1998, with the lowest values, and the other periods.

**Table 4:** Univariate PERMANOVA results of the Euclidean distance matrix based on Shannon diversity index. Bold values highlight statistical significance

Source	df	SS	MS	Pseudo-*F*	*P*	Unique perms
Zone	3	18.86	6.29	33.21	**0.000**	9959
Year	3	2.18	0.73	3.83	**0.024**	9962
ZoxYe	8	0.50	0.06	0.33	0.945	9945
Residuals	24	4.54	0.19			
Total	38	28.51				

**Fig. 4:**
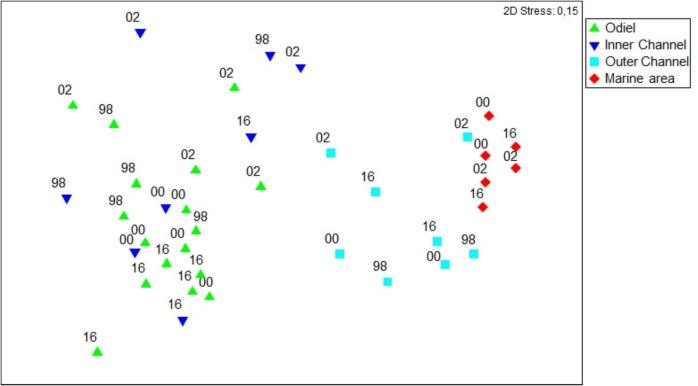
nMDS ordination using Bray–Curtis similarities on species abundance for all zones and sampling events

An nMDS analysis of macrobenthic assemblages over the entire study period showed similar trends to those described with the Shannon index (Fig. 4), displaying a gradient from upstream areas to the marine zone, regardless of the sampling period. The PERMANOVA test based on the Bray–Curtis similarity matrix showed significant spatial and temporal differences, but again no interaction effects (Table 5). The spatial post hoc test revealed significant differences among all the areas. Temporally, there were also significant differences in all years, except between 2000 and 2016.

**Table 5:** PERMANOVA and pairwise test results of the Bray–Curtis similarity matrix based on square-root-transformed data over sampling periods. In bold, significant results

Source	df	SS	MS	Pseudo-*F*	*p*	Unique perms
Zone	3	43638	14546	7.352	**0.001**	9902
Year	3	14226	4741.9	2.3967	**0.001**	9872
ZoxYe	8	19153	2394.1	1.2101	0.061	9777
Residuals	24	4.75E + 04	1978.5			
Total	38	1.29E + 05				
Pair-wise tests	*t*	*p*	Unique perms			
Zones						
Odiel, Inner Channel	1.4501	**0.020**	9935			
Odiel, Outer Channel	3.3091	**0.000**	9923			
Odiel, Marine	3.7718	**0.000**	9945			
Inner and Outer Channel	2.15	**0.003**	9914			
Inner Channel, Marine	2.6292	**0.003**	9940			
Outer Channel, Marine	1.7948	**0.012**	9931			
Years						
1998, 2000	1.5499	**0.007**	9923			
1998, 2002	1.8042	**0.001**	9933			
1998, 2016	1.4249	**0.027**	9926			
2000, 2002	1.7015	**0.001**	9934			
2000, 2016	1.2348	0.114	9928			
2002, 2016	1.6022	**0.005**	9925			

### Comparisons between biotic indices

The Pearson correlation values for the entire dataset (sampling events and stations) are presented in Table 6. *H*′, considered a reference index, showed the highest correlation with BENFES (0.916) and M-AMBI (0.639). BOPA and BO2A displayed a high correlation (0.919), which is expected as one is derived from the other. BENFES and M-AMBI also exhibited a strong correlation (0.692), as did AMBI and BENTIX (− 0.768).

**Table 6:** Pearson coefficients between indices over sampling periods and stations. H′ included as a reference. Significance level: ** (*p* < 0.01); * (*p* < 0.05)

	BOPA	BO2A	AMBI	M-AMBI	BENTIX	BENFES	H′
BOPA	1	0.919**	0.452**	− 0.501**	− 0.702**	− 0.498**	− 0.571**
BO2A		1	0.660**	− 0.576**	− 0.733**	− 0.564**	− 0.614**
AMBI			1	− 0.588**	− 0.768**	− 0.404*	− 0.391*
M-AMBI				1	0.665**	0.692**	0.747**
BENTIX					1	0.615**	0.639**
BENFES						1	0.916**
*H*′							1

**Fig. 5:**
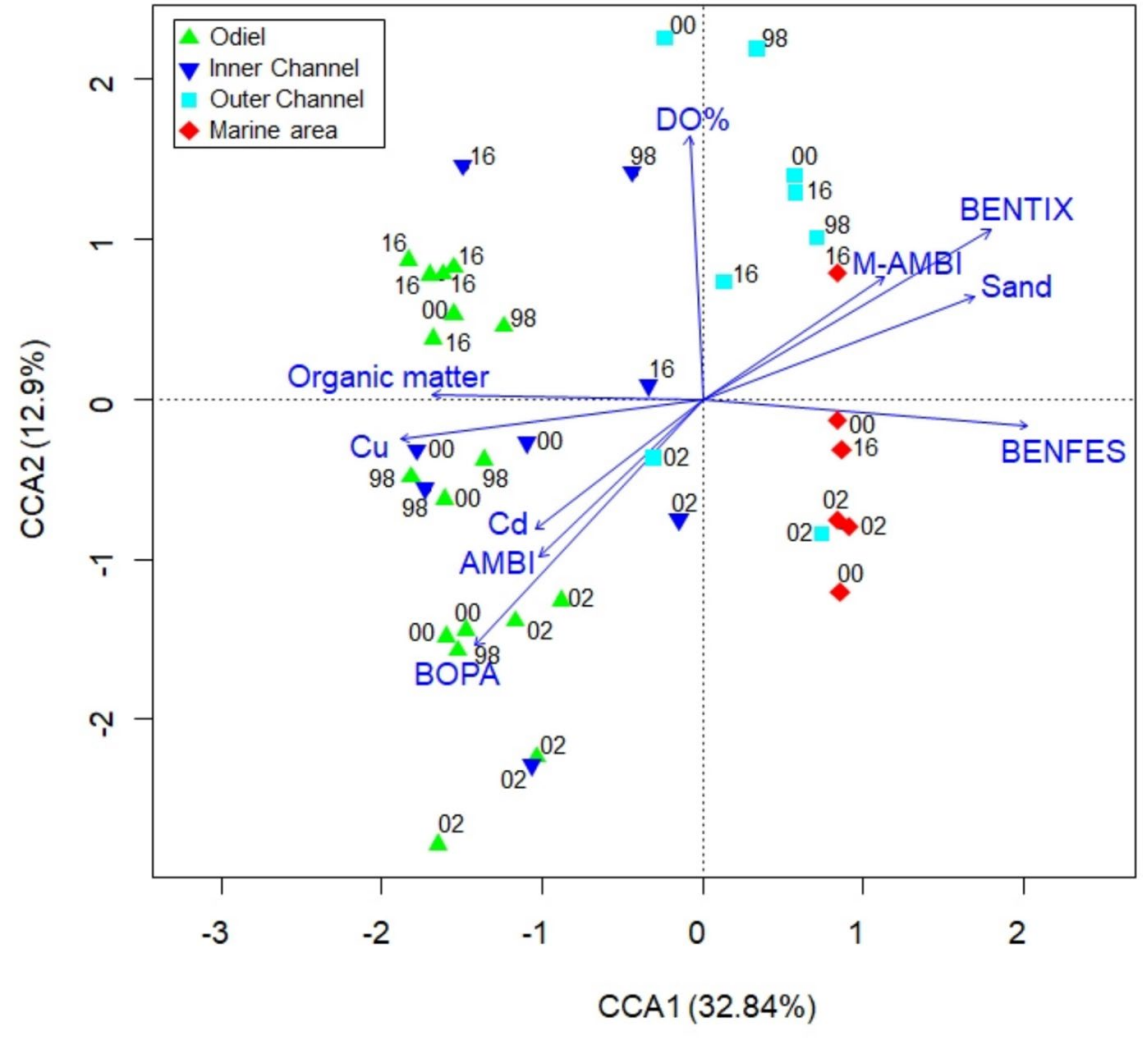
CCA analysis plot for all stations and sampling periods based on selected environmental variables and the biotic indices. The percentage of variability explained by the two principal axes is given

Canonical correspondence analysis (CCA) revealed a similar distribution of zones to that observed in the MDS, with a gradient from inner areas to the marine zone, regardless of the sampling period (Fig. 5). The first axis separated the zones according to the natural gradient from estuarine to marine environments. The permutation test identified Cu (*p* < 0.001), Cd (*p* < 0.01), and dissolved oxygen (*p* < 0.01) as the most influential environmental variables. Among the indices, BENFES and BOPA were the most significant (*p* < 0.001), followed by M-AMBI and BENTIX (*p* < 0.05), while AMBI was not significant (Table S3). In the outer zones, the environment was characterized by a higher percentage of sand and elevated values of M-AMBI, BENTIX, and BENFES, corresponding to better environmental conditions. In contrast, the inner zones were dominated by higher percentages of fine sediments, organic matter, and heavy metal concentrations, which were associated with higher values of AMBI and BOPA. BO2A was not included in the analysis due to collinearity with BOPA.

The average ecological quality status (EQS) for each index across the different areas and years is presented in Table 7 (individual index values for each sampling station and year are shown in Table S4). Overall, the M-AMBI and BENFES indices demonstrated a gradient of improving ecological status from the inner to the outer estuary zones. Poor and moderate EQS predominated in the inner zones, whereas good and high EQS were dominant in the outer zones. An improvement in EQS was observed by the 2016 sampling event, with inner zones transitioning from poor to moderate and outer zones consistently maintaining a high status. The BENTIX index showed a pattern similar to the other two indices but with greater temporal stability in the inner areas (remaining between poor and moderate) and a decline in the outer zones (from high to good). The AMBI index displayed a trend toward homogenization, with a predominance of good EQS across all areas. BOPA also exhibited a trend toward homogenization, showing moderate EQS in the inner zones, high EQS in the Outer Channel, and good EQS in the marine area. BO2A showed higher variability across all zones, ranging from moderate to bad EQS in the inner areas and moderate to good EQS in the outer zones. Slight improvements were observed in the inner zones (from poor to moderate) and a decline in the outer zones (from high to good).

**Table 7:**
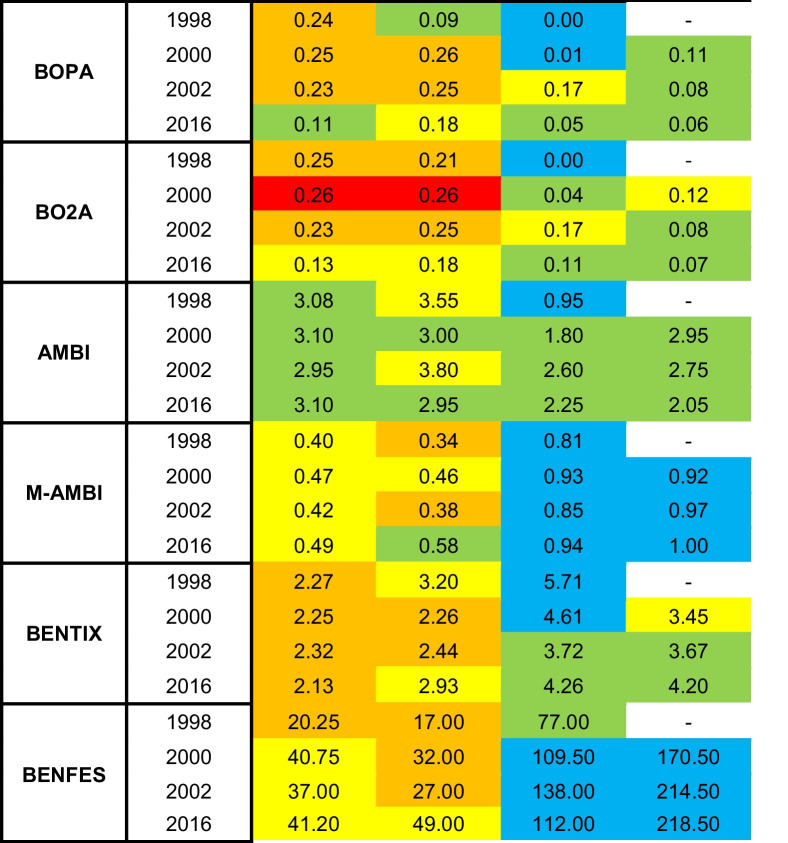
Average ecological quality status (EQS) and index values for each zone and year according to the results of the six biotic indices. Color code of EQS according to Table 1

PERMANOVA analysis (Table 8) revealed significant spatial and temporal differences for the BOPA, M-AMBI, and BENFES indices, while BENTIX showed a significant interaction between spatial and temporal factors. AMBI and BO2A exhibited significant spatial differences only. In pairwise comparisons (Table S5), BOPA, BO2A, AMBI, and M-AMBI grouped the zones into two homogeneous clusters: Odiel + Inner Channel and Outer Channel + Marine Area. BENFES also grouped the inner zones into a single cluster but distinguished between the Outer Channel and Marine Area. Temporally, BOPA did not show a clear pattern, although the main difference was observed between 1998 and most other sampling events, except for 2016, where a trend toward EQS homogenization across the study area was noted. M-AMBI identified 2016 as distinct from the other years, primarily due to the improvement in EQS in the inner zones, which shifted from poor-moderate to good. Similarly, BENFES differentiated 1998 from other sampling events, showing a transition from poor EQS in the inner zones and good EQS in the outer zones to moderate EQS in the inner zones and high EQS in the outer zones. The significant interaction for BENTIX was attributed to spatial differences across sampling events. In 1998, all areas were distinct. In 2000 and 2002, contiguous areas formed homogeneous groups (Odiel + Inner Channel, Inner Channel + Outer Channel, and Outer Channel + Marine Area). By 2016, only the Odiel zone remained distinct, while the outer zones transitioned from high to good EQS.

**Table 8:** Univariate PERMANOVA results based on the Euclidean distance matrix of the six biotic indices. In bold, significant results

Source	df	SS	MS	Pseudo-*F*	*p*	Unique perms
BOPA						
Zone	3	0.17747	5.92E − 02	13.168	**0.0001**	9963
Year	3	5.14E − 02	1.71E − 02	3.8145	**0.0232**	9951
ZoxYe	8	7.83E − 02	9.79E − 03	2.1787	0.0683	9949
Residuals	24	0.10782	4.49E − 03			
Total	38	0.40404				
BO2A						
Zone	3	0.16845	5.62E − 02	11.536	**0.0001**	9938
Year	3	2.47E − 02	8.22E − 03	1.6886	0.1974	9955
ZoxYe	8	5.52E − 02	6.90E − 03	1.4184	0.2379	9937
Residuals	24	0.11681	4.87E − 03			
Total	38	0.36486				
AMBI						
Zone	3	10.282	3.4272	6.4298	**0.0017**	9971
Year	3	1.4636	0.48785	0.91526	0.4525	9952
ZoxYe	8	4.2131	0.52664	0.98803	0.4714	9952
Residuals	24	12.793	0.53302			
Total	38	27.95				
M-AMBI						
Zone	3	1.0772	0.35906	15.697	**0.0001**	9949
Year	3	0.30852	0.10284	4.4959	**0.0108**	9951
ZoxYe	8	0.23987	3.00E − 02	1.3108	0.301	9942
Residuals	24	0.54897	2.29E − 02			
Total	38	2.1865				
BENTIX						
Zone	3	35.241	11.747	69.764	**0.0001**	9940
Year	3	3.5121	1.1707	6.9527	**0.0016**	9954
ZoxYe	8	3.8653	0.48316	2.8694	**0.0211**	9950
Residuals	24	4.0412	0.16838			
Total	38	44.133				
BENFES						
Zone	3	1.32E + 05	43,988	79.28	**0.0001**	9962
Year	3	5662.2	1887.4	3.4017	**0.033**	9964
ZoxYe	8	3942	492.75	0.88808	0.5455	9941
Residuals	24	13,316	554.85			
Total	38	1.69E + 05				

The highest Kappa values (very good agreement) were obtained between BENFES and M-AMBI (*p* < 0.0001) (Table 9). In general, both indices also showed good agreement with BOPA, BO2A, and BENTIX (*p* < 0.0001). The greatest disagreement was found for AMBI (very poor with BOPA, BO2A, and BENFES; and poor with M-AMBI and BENTIX).

**Table 9:**
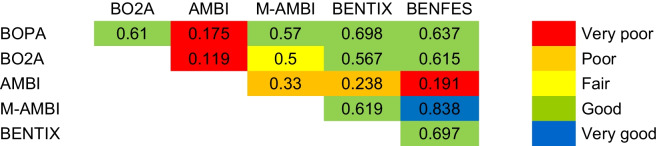
Kappa values and agreement levels (in color code) for the ecological quality status (EQS) between each index. Level of agreement “null,” “excellent,” and “perfect” were not found

## Discussion

The evaluation of ecological quality in marine and estuarine ecosystems has become a priority in many countries due to climate change and anthropogenic activities that negatively impact the environment. These growing environmental pressures demand the implementation of scientifically based conservation strategies and appropriate environmental management measures (Anaisce et al., 2023). In this context, our study provides a comprehensive assessment of the long-term effectiveness of corrective measures implemented in the Odiel Estuary and their influence on ecological quality, as evaluated using six biotic indices. Our findings confirm the expected spatial gradient along the estuary over an 18-year period, characterized by improving environmental conditions from the inner zones toward the marine area. Additionally, temporal trends indicate a gradual but slight improvement in water quality and benthic community structure, particularly evident in the 2016 sampling event.

A clear indication that the Odiel Estuary is still subject to elevated levels of disturbance is its consistently lower species richness and diversity compared to other well-mixed estuaries in the region, especially in the inner zones. For example, the average Shannon diversity index (H') values in the Odiel and Inner Channel remained around 1.4 throughout the study period, while values in comparable stretches of the Guadiana Estuary (located westward, near the Portuguese border) exceeded 2.5, and those in the Guadalquivir Estuary (to the east) reached approximately 2.4 (Sánchez-Moyano et al., 2017). Similar diversity levels have been reported in the Palmones Estuary, located in the Strait of Gibraltar, with *H*′ values also around 2.5 (Estacio et al., 1999). Furthermore, recent findings from Sánchez-Moyano et al. (2025) on crustacean assemblages support this pattern, showing that species richness in the Odiel Estuary was lower than in other well-mixed estuaries of the Gulf of Cádiz (e.g., Guadalquivir, Guadiana, or Piedras), and more comparable to values observed in the highly polluted Mondego Estuary in central Portugal.

The macrobenthic community structure exhibited a strong spatial gradient, with increased species diversity and abundance toward the marine area. This pattern aligns with the expectation that inner estuarine zones, which experience greater environmental fluctuations and higher levels of contamination, support communities that are more tolerant of pollution but lower in diversity (Dauvin, 2008; Tweedley et al., 2015). This finding is consistent with previous taxonomic studies in the area, both at the family level (Sánchez-Moyano et al. 2010) and in crustacean assemblages (Sánchez-Moyano & García-Asencio, 2010; Sánchez-Moyano et al. 2015).

According to Pelletier et al. (2018), historical data are essential for inferring environmental changes. Unfortunately, only one study was conducted in 1981, prior to the first pollution control program in 1986 (Cano & García, 1987). Furthermore, comparisons are challenging because that study was conducted in winter, a period typically associated with lower diversity and reduced community development in temperate zones (Boesch et al., 1976; Vinagre et al., 2015; Ysebaert & Herman, 2002), and was based on a small number of samples with limited spatial coverage of the estuary, except in the adjacent marine area. However, Cano and García (1987) reported severe environmental degradation in 1981, with only two species recorded in the innermost estuary (the highly abundant polychaete *Hediste diversicolor*, ca. 1000 individuals/m^2^), four species in the Outer Channel (mainly the polychaetes *H. diversicolor* and *Nephthys* spp. and the crustacean *Cyathura carinata*), and just 30 species in low abundance (with a maximum of ca 50 individuals/m^2^, including hermit crabs and the bivalve *Chamelea gallina*) in the marine area. By contrast, our study revealed temporal changes in the estuary, including a higher total and per-species abundance compared to the 1981 study (see Table S2) and a gradual increase in diversity from the 1998 to the 2016 sampling events. The significant increase in species diversity in 2016 suggests a positive response of benthic assemblages to improving environmental conditions. However, a longer-term monitoring plan is necessary to determine whether this trend reflects the effectiveness of corrective measures or simply the natural fluctuations of dynamic estuarine environments.

Similarly, the observed trends in environmental parameters exhibited the same spatial pattern throughout the study period, with a higher percentage of organic matter and heavy metal concentrations in the inner estuary and a predominance of coarse sands and dissolved oxygen in the outer zones. Salinity, although considered one of the main factors influencing the composition of macrobenthic communities in estuaries (Gomes et al., 2024; Van Diggelen & Montagna, 2016), was very similar throughout the study area and did not show a clear gradient, primarily because the study focused on the euhaline zone, which is strongly influenced by marine conditions. The high concentrations of heavy metals such as Zn and Cu recorded in the inner estuarine zones confirm the persistence of contamination despite remediation efforts. Although the variations in sediment grain size and organic matter content follow the natural estuarine pattern (Elliott & Whitfield, 2011; McLusky & Elliott, 2004), the elevated metal concentrations in the inner estuary are directly linked to historical acid mine drainage and industrial activities (Nieto et al., 2013; Olías et al., 2006; Pérez-López et al., 2011, 2016; Sarmiento et al., 2009).

The comparative analysis of biotic indices provided critical insights into their applicability for assessing ecological quality in the Odiel Estuary. One of the major challenges in estuarine ecological assessment is distinguishing between natural and anthropogenic disturbances (Birk et al., 2012; Elliott & Quintino, 2007). Given the naturally stressed conditions of estuaries, it is essential to validate biotic indices across different environmental contexts to ensure their reliability (Mulik et al., 2020). In this regard, the PERMANOVA results confirmed the ability of most indices to detect both spatial and temporal differences in ecological quality between inner and outer estuarine areas. The absence of a significant interaction between zone and year, despite significant main effects for both factors, suggests a temporally consistent spatial pattern, accompanied by an overall improvement in ecological conditions across all zones.

Overall, the Odiel and Inner Channel zones predominantly exhibited poor and bad ecological status, with some indices (e.g., M-AMBI, BOPA, BENFES) showing slight temporal improvements, while others remained relatively stable throughout the study period (e.g., AMBI, BENTIX). Meanwhile, the Outer Channel and Marine areas consistently presented high ecological quality values, maintaining temporal stability. Regarding kappa values, a high concordance was observed between BENTIX, BENFES, and M-AMBI, whereas AMBI, BOPA, and BO2A showed lower agreement. The discrepancies among indices reinforce the argument that no single index is universally reliable, and a multi-index approach enhances the robustness of ecological assessments (Afli et al., 2008; Dauvin et al., 2012; Salas et al., 2006), particularly in highly dynamic and impacted systems like the Odiel Estuary.

M-AMBI has been considered one of the most suitable indices for assessing the integrity of marine and coastal ecosystems (Borja et al., 2015; Cai et al., 2015; Anasice et al. 2023). It integrates AMBI, species richness, and Shannon diversity and can respond to different types of environmental pressures, such as urban and industrial discharges, dredging and sediment disposal, and engineering works (Borja et al., 2009; Costa-Dias et al., 2010; Muxika et al., 2007). This index, characterized by its intuitive nature, facilitates understanding among benthic ecologists and managers, as it is based on reference conditions. This allows for evaluating how far an aquatic system is from achieving its ecological objectives while demonstrating greater sensitivity to anthropogenic pressures than to natural variability, which is crucial in aquatic systems with high seasonal variability, such as estuaries (Borja & Tunberg, 2011). However, the need to establish these reference conditions for M-AMBI computation complicates its application (Mulik et al., 2020), and if incorrect values are used, the index results may be inaccurate (Brauko et al., 2016; Hutton et al., 2015; Munari & Mistri, 2010). In the present study, M-AMBI and BENFES exhibited the strongest correlations with Shannon diversity and effectively captured the spatial and temporal gradients of environmental quality, suggesting their suitability for monitoring transitional ecosystems. Several comparative studies evaluating the efficacy of different indices in various parts of the world confirm the robustness of M-AMBI (Puente, and Diaz, 2008; Nebra et al., 2014; Sivaraj et al., 2014; Pitacco et al., 2018; Dong et al., 2023; Song et al., 2023; Santibañez-Aguascalientes et al., 2023).

AMBI is also widely used for assessing the environmental quality of coastal systems (Borja et al., 2007, 2015; Mulik et al., 2017; Saracho Bottero et al., 2020). However, some authors have pointed out its lack of sensitivity in differentiating between contaminated areas where naturally stressed communities predominate, due to the dominance of tolerant species commonly found in such environments (Li et al., 2021; Munari & Mistri, 2010; Puente et al., 2008). AMBI tends to homogenize ecological quality status across zones, potentially underestimating the extent of degradation in inner areas. Similar concerns have been raised in other geographical regions (Jayachandran et al., 2024; Neves et al., 2025; Pitacco et al., 2018; Sigamani et al., 2015; Tweedley et al., 2015).

BENTIX, derived from AMBI, improves its efficiency by using only three ecological groups instead of five (Simboura & Argyrou, 2010). It appears to be more sensitive than AMBI to organic matter increases in sediments (Afli et al., 2008), possibly because in BENTIX, each ecological group is weighted equally, whereas in AMBI, each has a different coefficient (Blanchet et al., 2008; Hutton et al., 2015). However, in some cases, this can lead to discrepancies in taxon sensitivity when assigning species to ecological groups. A species may be classified as tolerant (group II) in BENTIX, while AMBI places it in the sensitive group (group I) (Dias et al., 2018). In our study, BENTIX exhibited minimal temporal changes and high homogeneity in the innermost zones, while displaying a lower ecological status than M-AMBI and BENFES in the outer zones. Underestimation of ecological status and poor discriminating power have been reported for this index, particularly in transitional waters (Garaffo et al., 2017; Magni et al., 2023; Mulik et al., 2020; Pitacco et al., 2018).

BOPA and BO2A are less suitable for estuarine zone assessments, as their calculation is based solely on the presence of opportunistic and sensitive species (such as annelids and polychaetes) without considering other equally important macrobenthic taxa (Dias et al., 2018; Mulik et al., 2020). They also show low discriminatory ability and a tendency to overestimate ecological status (Dong et al., 2023; Nebra et al., 2014). Additionally, some studies indicate that sensitivity to pollution can vary between species, responding to stressors differently than expected (Afli et al., 2008). Other studies highlight that these indices are not applicable in sites with low individual counts, as some stations may lack the macrobenthic species required for their calculation (Dauvin & Ruellet, 2009), as observed in certain stations of the Odiel Estuary. BOPA and BO2A, which exhibited a strong correlation with each other, provided a more nuanced assessment of ecological quality but showed higher variability in their responses.

Finally, BENFES has proven effective in detecting changes or variations in environmental quality. Additionally, its calculation is straightforward and cost-effective, as it is based on the presence or absence of organisms at the family level, reducing errors associated with taxonomic identification (Sánchez-Moyano et al., 2017). Furthermore, BENFES exhibited the highest concordance with M-AMBI among all the indices studied. Although BENFES does not meet the requirements of the Water Framework Directive due to its family-level assessment, it can be useful as a preliminary method and for long-term environmental quality monitoring, providing insights into system evolution. A more detailed species-level study would only be required in cases of significant changes.

In summary, the improvement in ecological quality observed in 2016, particularly in the inner estuary, suggests that long-term remediation efforts have contributed to some degree of ecosystem recovery. However, persistent contamination levels and the continued degradation of certain biological indicators highlight the need for ongoing monitoring and adaptive management strategies. Hence, the results of this study have important implications for the development of monitoring programs and management strategies in estuarine environments. Given M-AMBI’s demonstrated effectiveness in detecting spatial and temporal changes in ecological quality—due to its ability to integrate multiple aspects of biodiversity and its sensitivity to various anthropogenic pressures—alongside BENFES’s utility as a long-term environmental quality monitoring method due to its high correlation with M-AMBI and rapid execution, these indices should be prioritized in routine assessments of transitional waters. Additionally, BENTIX may serve a secondary role in these assessments. Furthermore, the observed improvements in ecological quality underscore the value of long-term environmental policies and remediation efforts, emphasizing the need for continued investment in pollution control and habitat restoration. Future research should focus on refining the calibration of biotic indices for estuarine environments and exploring complementary approaches, such as functional trait analyses, to enhance the resolution of ecological assessments.

Finally, to assist future practitioners, a decision-support table outlines the key strengths, limitations, and optimal usage scenarios of the six biotic indices evaluated in this study (Table 10).

**Table 10:** Decision table for selecting biotic indices in transitional waters based on performance, applicability, and context-specific considerations

Index	Strengths	Limitations	Recommended use
M-AMBI	Integrative (taxa, diversity, sensitivity), sensitive to pressures, widely validated, high concordance	Requires accurate reference conditions; more complex calculation	Primary index for detecting spatial–temporal gradients and evaluating management outcomes
BENFES	Cost-effective, based on presence/absence, high correlation with M-AMBI, quick to apply	Family-level resolution; not compliant with WFD requirements	Complementary or preliminary index for long-term monitoring or rapid assessments
BENTIX	Simplified AMBI; equal weight for groups; sensitive to organic enrichment	May underestimate status in outer zones; lower taxonomic resolution; some species misclassified	Secondary index for general trends, especially in moderately impacted areas
AMBI	Widely used, references available, simple to calculate	Can homogenize status; low sensitivity in stressed systems dominated by tolerant species	Supplementary tool; use cautiously in estuaries with strong natural variability or tolerant-dominated assemblages
BOPA/BO2A	Focused on opportunists vs sensitive species; quick calculation	Excludes many taxa; less reliable in low-density stations; overestimate status	Not recommended as standalone tools; limited use in screening or as supporting metrics

## Conclusion

This study provides robust evidence of a spatial and temporal gradient in ecological quality in the Odiel Estuary, with detectable improvements observed following the implementation of corrective measures. These findings underscore the need to enhance environmental management strategies and improve the efficiency of decontamination processes in the region to mitigate pollution impacts and promote biodiversity recovery in affected areas. The results highlight the utility of a multi-index approach for assessing estuarine health and emphasize the importance of continued monitoring efforts to track environmental changes. Hence, we propose a monitoring routine primarily based on the use of BENFES as a rapid and cost-effective assessment method for evaluating the ecological status of transitional and coastal waters. While BENFES does not fully comply with the Water Framework Directive (WFD) requirements regarding the use of species abundance and richness data, and, therefore, should not be used as a standalone regulatory index, it offers a practical and efficient tool for long-term monitoring. Therefore, we recommend its application as a complementary or preliminary tool, particularly useful for identifying broad-scale trends or potential disturbances. When significant environmental changes are detected, more comprehensive indices such as M-AMBI—which require detailed taxonomic resolution and abundance data—should be applied. Ultimately, these results support the broader application of biotic indices for evaluating ecosystem health in transitional waters worldwide, contributing to the development of more effective management and conservation strategies.

## Supplementary Information

Below is the link to the electronic supplementary material.

Supplementary file1 (XLSX 59 KB)

## Data Availability

The main data supporting the findings of this study are available within the paper and its Supplementary Information. Other data will be made available on request.
